# Supplementary figures and images for: Genome-wide meta-analysis identifies genetic risk loci for mono- and polyneuropathies in 983 477 individuals
Source: Hum Mol Genet. 2026 Jan 10;35(4):ddaf200. doi: 10.1093/hmg/ddaf200 (PMC13036838; doi:10.1093/hmg/ddaf200)

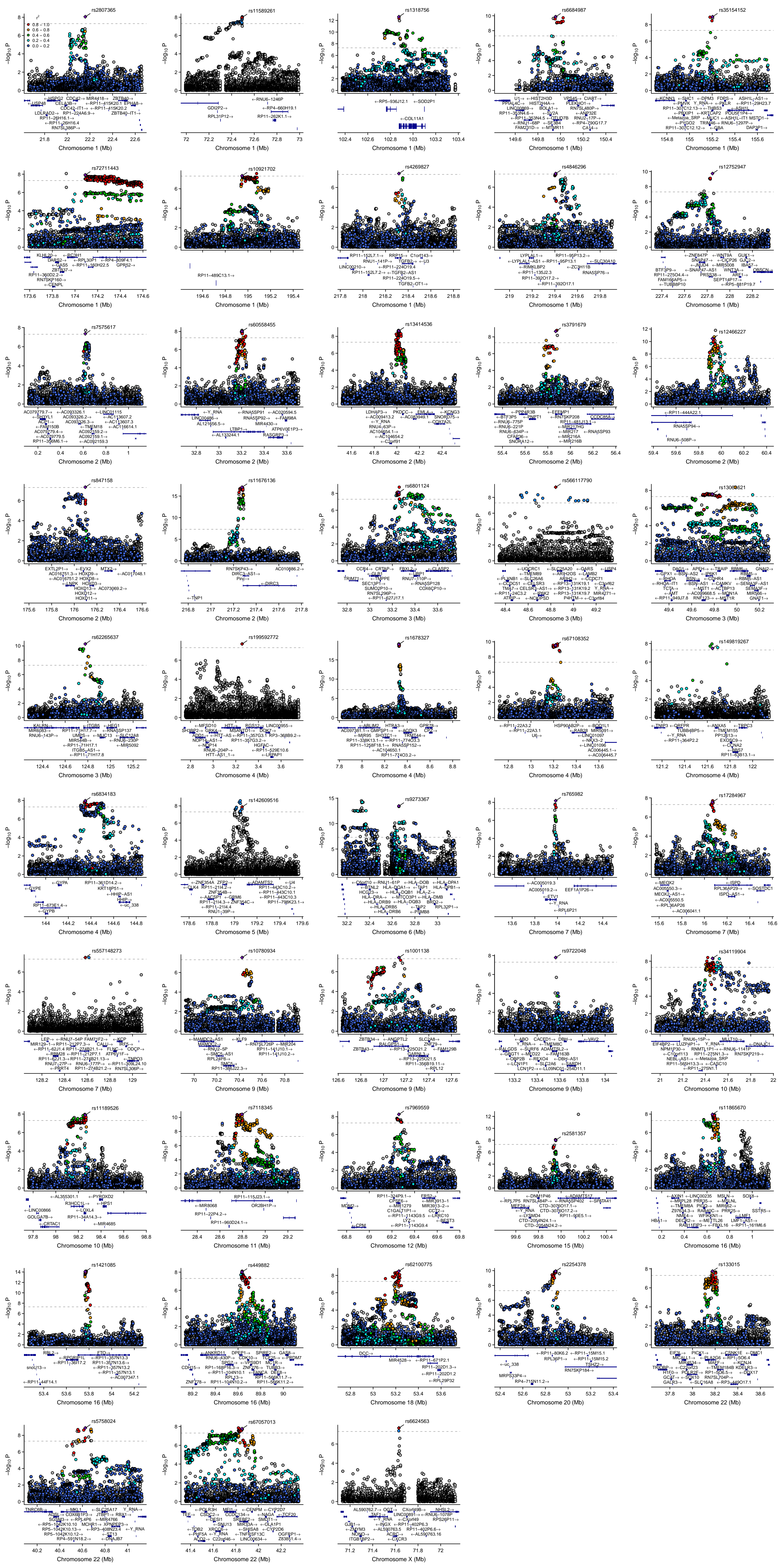

Supplement: Supplemental_Figure_S1_ddaf200 [file supplemental_figure_s1_ddaf200.pdf]

Neuropathies meta analysis qq plot

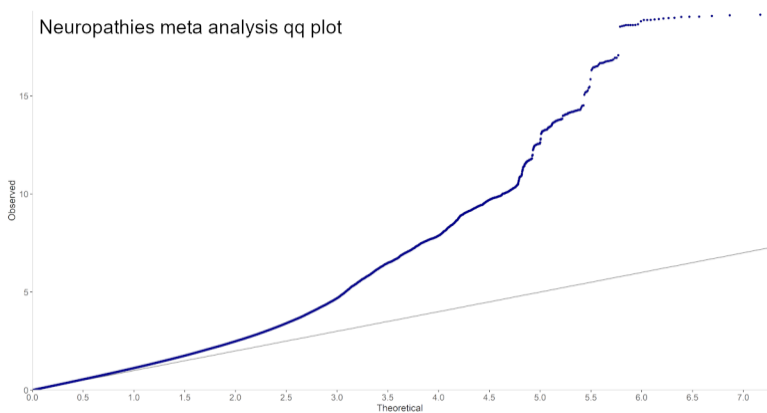

Supplement: Supplemental_Figure_S2_ddaf200 [file supplemental_figure_s2_ddaf200.pdf]

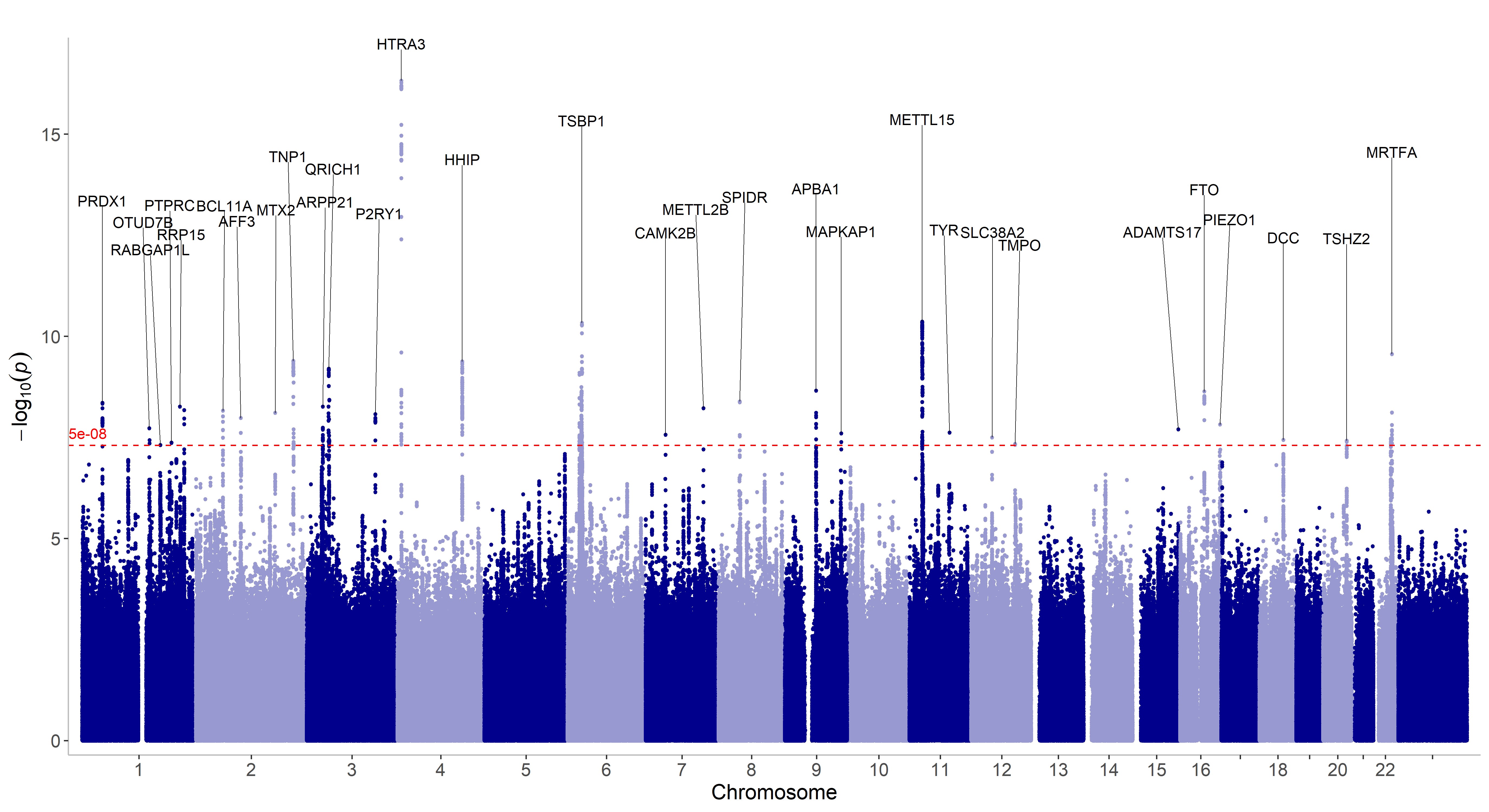

Supplement: Supplemental_Figure_S3_ddaf200 [file supplemental_figure_s3_ddaf200.jpeg]

# FinnGen

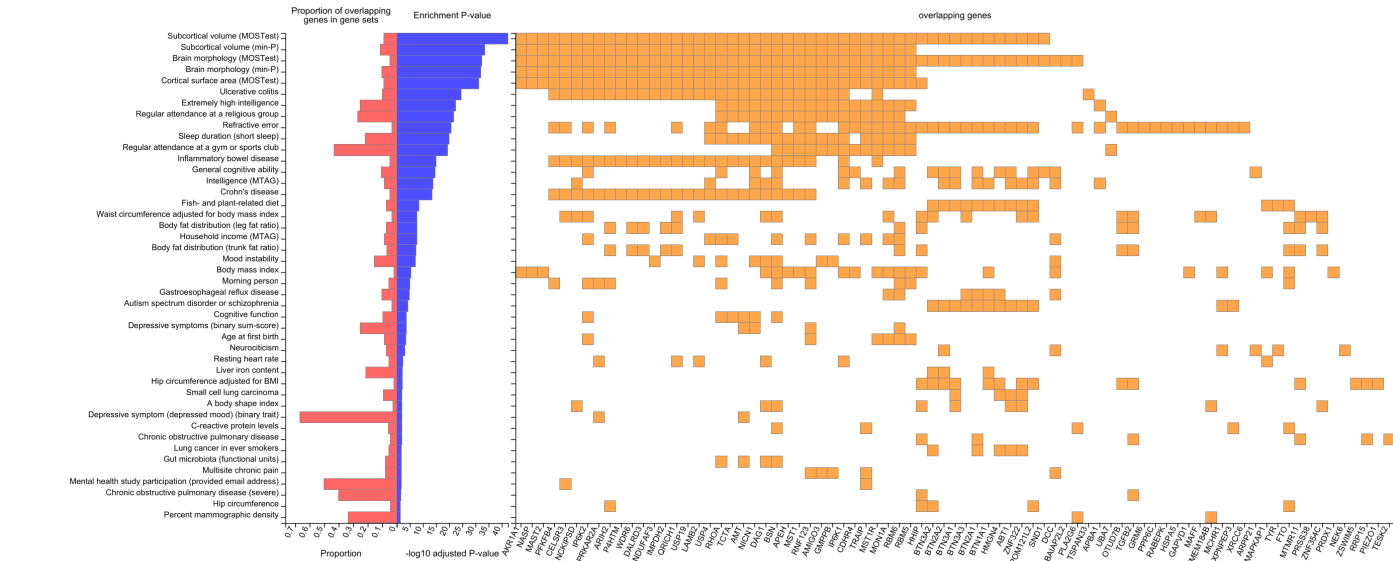

# UKB

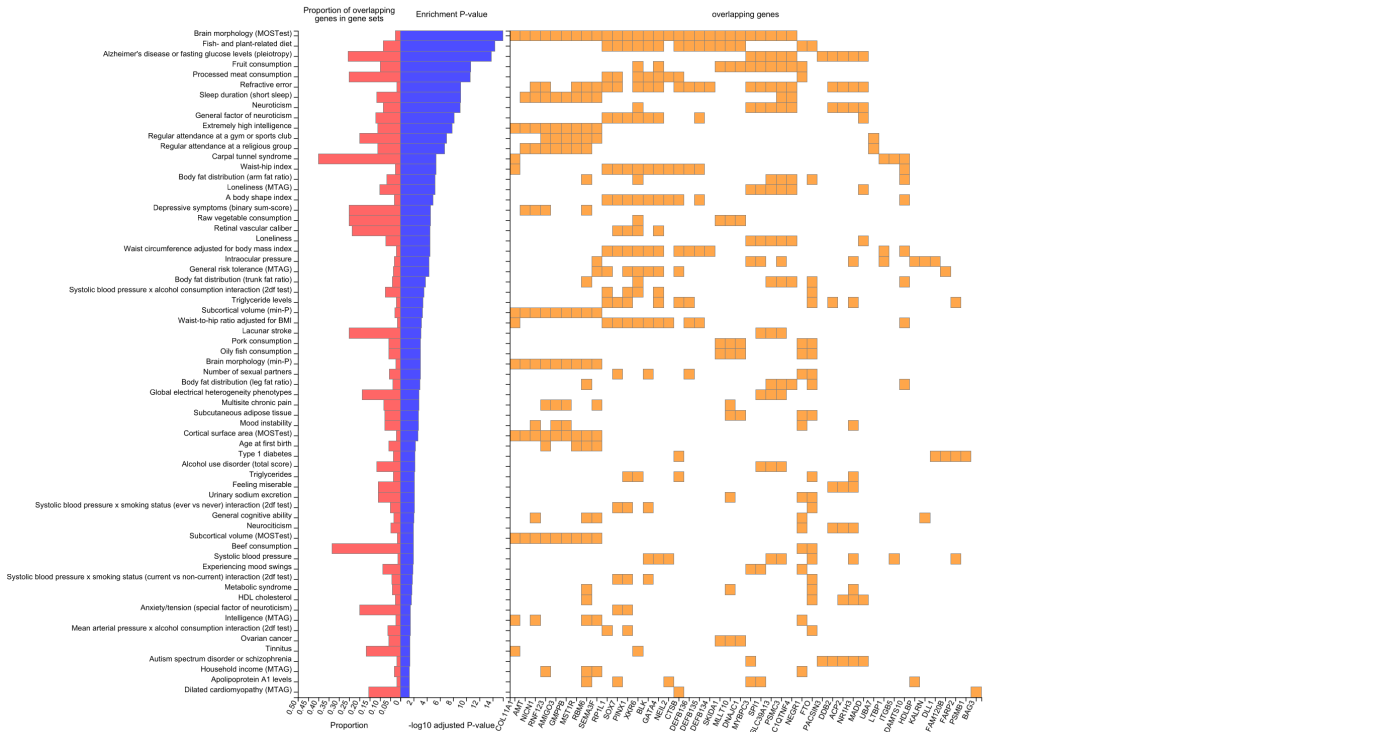

Supplement: Supplemental_Figure_S4_ddaf200 [file supplemental_figure_s4_ddaf200.pdf]
